# Ameliorating effects of bromelain with or without metformin on endocrine-metabolic disturbances in letrozole-induced polycystic ovary syndrome in female rats via targeting SIRT1, insulin resistance, and inflammatory axis

**DOI:** 10.1007/s00210-025-04517-w

**Published:** 2025-08-30

**Authors:** Marwa S. Arab, Dina M. Tahoon, Amira A. El Saadany, Sabeha E. Hedya

**Affiliations:** Pharmacology Department, Faculty of Medicine, Tanta University, Tanta, 31527 Egypt

**Keywords:** PCOS, Letrozole, Bromelain, Metformin, SIRT1, Insulin resistance, Inflammation

## Abstract

Polycystic ovary syndrome (PCOS) is the most prevalent endocrinopathy affecting women in the reproductive age range, which is thought to be the primary cause of anovulatory infertility. This condition is complex and heterogeneous. The current study is aimed at determining the potential ameliorative effects of bromelain and metformin, either separately or in combination, in the reduction of letrozole-induced PCOS in female rats by focusing on the inflammatory axis, homeostasis model assessment of insulin resistance (HOMA-IR), and silencing information regulator 2-related enzyme 1 (SIRT1). The study was performed on 48 female albino rats divided into 6 groups (control, vehicle, untreated PCOS, PCOS + metformin, PCOS + bromelain, combined), vaginal smear was done to evaluate ovarian cyclicity and biochemical parameters, and the histopathological picture of ovarian tissue was assessed. Administration of either bromelain or metformin revealed significant reduction in ovarian weight (OW) and serum levels (testosterone, luteinizing hormone (LH)) and improvement of vaginal smear and histopathological picture, bromelain revealed more significant reduction of body weight (BW), serum level of follicular stimulating hormone, and tissue level of tumor necrosis factor α (TNFα), while metformin revealed more significant reduction of insulin resistance (IR) by assessment of HOMA-IR and increase tissue level of SIRT1, while the values of BW (*p* < 0.001), OW (*p* < 0.01), testosterone (*p* < 0.05), LH (*p* < 0.05), fasting glucose (*p* < 0.01), fasting insulin (*p* < 0.01), HOMA-IR (*p* < 0.01), and TNFα (*p* < 0.001) decreased in bromelain treated group compared to diseased group; the values of FSH/LH ratio (*p* < 0.05) and SIRT1 (*p* < 0.05) increased; and these advantageous benefits were markedly more pronounced in rats administered the combined treatment compared to monotherapy.

**Supplementary Information:**

The online version contains supplementary material available at 10.1007/s00210-025-04517-w.

## Introduction

PCOS is a prevalent endocrinopathy affecting 4 to 21% of reproductive-aged women (Ozegowska et al. 2020). It accounts for roughly 70–80% of affected women and is thought to be the primary cause of anovulatory infertility (Collée et al. 2021).

SIRT1 has been shown to protect against PCOS by ameliorating mitochondrial abnormalities and lowering the expression of oxidative stress indicators (Di Emidio et al. 2019). Activation of SIRT1 inhibits androgen receptor expression and alters the structural fibrosis of the ovary (Wang et al. 2020).

The IR results in androgen-dependent anovulation through various mechanisms; insulin amplifies the stimulatory effects of LH on androgen synthesis in ovarian theca cells (Unluhizarci et al. 2021).

Hyperandrogenism promotes the differentiation of pre-adipocytes into adipocytes, particularly in the abdominal region, resulting in the emergence of visceral obesity (Amiri et al. 2021), which is marked by a chronic inflammatory state (Khanna et al. 2022).

PCOS can be triggered by elevated androgen levels, which are the primary catalyst for the manifestation of its signs and symptoms (Gao et al. 2020).

Letrozole is a non-steroidal aromatase inhibitor utilized in the treatment of estrogen-dependent breast cancer (Sayyad et al. 2022). Letrozole inhibits the conversion of testosterone to estradiol, resulting in elevated androgen levels both systemically and intraovarian (Younas et al. 2022). This hormonal imbalance manifests as polycystic ovaries, follicular atresia, and aberrant follicular development (Nallathambi and Bhargavan 2019).

Metformin is an insulin sensitizer drug that is used as an oral antihyperglycemic agent for type 2 diabetes mellitus (Abdalla et al. 2022) because it can lower IR, BW, and testosterone levels, promote more regular menstrual cycles and ovulation, and treat PCOS (Tao et al. 2021).

As many of the medicines used in the treatment of PCOS have adverse effects that restrict their use for extended periods, this necessitates searching for alternative natural agents with low side effects.

Bromelain, which is naturally present in pineapple plants, was chosen because it possesses anti-inflammatory qualities by lowering the production of pro-inflammatory molecules like TNFα (Alves Nobre et al. 2025), it induces SIRT1 protein expression which regulates mitochondrial function and insulin signaling (Iside et al. 2020), and it has also been shown to possess anti-hyperglycemic properties (Alves et al. 2020). Furthermore, pineapple has emerged as a beneficial fruit in treating obesity and dyslipidemia due to its high bromelain content, which contributes to lipolysis and alleviates the severity of cardiovascular conditions (Chen et al. 2022).

Therefore, this study sought to determine the potential ameliorative effects of bromelain and metformin, either separately or in combination, in reducing letrozole-induced PCOS in female rats by focusing on the inflammatory axis, HOMA-IR, and SIRT1.

## Materials and methods

### Ethical considerations

All experimental methods were conducted in the Medical Pharmacology Department of the Faculty of Medicine, Tanta University, Egypt. Animal handling and experimental procedures adhered to the rules established by the Research Ethics Committee of the Faculty of Medicine, Tanta University, Egypt (approval no. 36264MS343/10/23).

### Drugs and chemicals used

Letrozole, a crystalline white to yellow powder from Acros Organics Company (catalog no. 463190010), was dissolved in 0.5% carboxymethylcellulose (CMC). Metformin, supplied from Glucophage 1000 mg tab (Minapharm Company for Pharmaceuticals and Chemical Industries, Cairo, Egypt), was crushed and dissolved in 0.5% CMC. Bromelain, a light yellow to brown powder from Solarbio Company (catalog no. 9001–00-7), was dissolved in normal saline.

### Animals

The research involved 48 female Wistar Albino rats weighing approximately 150–200 g. Rats were housed in wire mesh cages under strict hygienic conditions and given unrestricted access to a normal animal meal and water for the experiment.

The required sample size (*n*) was calculated using the Resource Equation Method, which balances statistical power with ethical animal use. For 6 experimental groups (*G* = 6) and a target error degree of freedom (*E* = 20), *N* = *E* + *G* = 26. To account for 20% potential attrition and the potential use of non-parametric tests (e.g., Kruskal–Wallis), an additional sample size was required due to lower statistical power. So, a further adjustment was made. The final sample size of 42 animals (7 per group) allows for dropouts and ensures sufficient sensitivity to detect biologically relevant differences, and we added one to each group to compensate for the loss regarding to pilot study.

### Randomization and blindness

After acclimatization, rats were randomized using a computer-generated sequence (www.randomizer.org). Outcome assessors (histopathology, vaginal cytology) were blinded to treatment groups. ELISA plate readers were calibrated by technicians unaware of the sample origins.

### The experimental protocol

Animals were permitted a 2-week acclimatization period and were randomly assigned to 6 equal groups, with 8 rats in each group, after assessing ovarian cyclicity by vaginal smear.

(i) Control group: rats served as the normal control group.
(ii) Vehicle group: received CMC in a dose of 0.5 ml/rat/day by oral gavage once daily for 5 weeks (Morsi et al. 2022).
(iii) Untreated PCOS group: received letrozole in a dose of 1 mg/kg/day by oral gavage once daily for 21 consecutive days (Ibrahim et al. 2022).
(iv) Metformin-treated PCOS group: received letrozole 1 mg/kg/day by oral gavage once daily for 21 consecutive days, then metformin was given by oral gavage in a dose of 500 mg/kg/day (Ndeingang et al. 2019) for 14 days from the 22nd day till the 35th day.
(v) Bromelain-treated PCOS group: received letrozole 1 mg/kg/day by oral gavage once daily for 21 consecutive days, then bromelain was given by oral gavage in a dose of 40 mg/kg/day (Murad et al. 2019) for 14 days from the 22nd day till the 35th day.
(vi) Combined group: received letrozole 1 mg/kg/day by oral gavage once daily for 21 consecutive days, then metformin in a dose of 500 mg/kg/day and bromelain in a dose of 40 mg/kg/day by oral gavage for 14 days from the 22nd day till the 35th day.

**Fig. 1:**
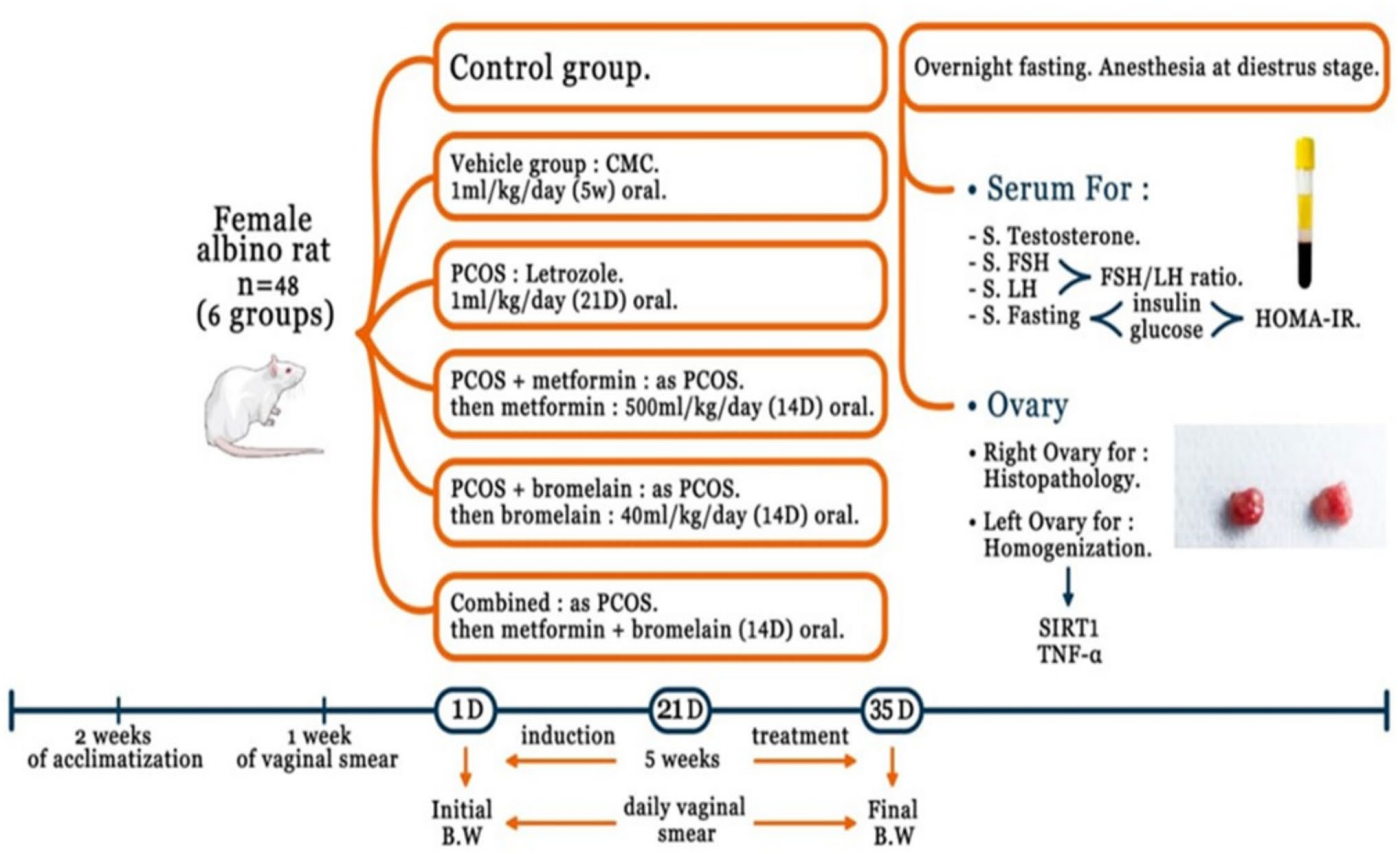
Schematic illustration of the experimental design of the study

The BW of rats was recorded at the beginning (initial) and at the end (final) of the experiment (Fig. 1).

### The dose justification

The selected doses, based on prior evidence of efficacy in rodent disease models, were chosen to ensure biological relevance, maximize translational potential, and uphold ethical standards in preclinical research. Metformin dose (500 mg/kg/day) was selected based on (Ndeingang et al. 2019), and bromelain dose (40 mg/kg/day) was selected based on (Murad et al. 2019). Using FDA guidance for human equivalent dose (HED) calculation based on body surface area [HED (mg/kg) = Animal dose (mg/kg) × (Animal Km/Human Km) For rats to humans: HED = Animal dose × (6/37)], the metformin dose equates to ~ 81 mg/kg (5.7 g/day) in humans—demonstrating alignment with clinical PCOS treatment ranges (1.5–3.0 g/day). Similarly, the bromelain dose translates to ~ 6.5 mg/kg (455 mg/day), within the 200–800 mg/day clinical range.

### Vaginal smear

All animals had daily vaginal smears to assess ovarian cyclicity; those exhibiting regular estrous cycles were selected for inclusion in the study. Vaginal smears were obtained using cotton swabs moistened with saline, which were gently inserted into the vagina. The collected smears were then placed on glass slides, dried, fixed, and stained with metachromatic hematoxylin and eosin (H&E) (Merkwitz et al. 2016). Cycles lasting 4 to 5 days were deemed regular, and estrus stages were identified through the analysis of vaginal smears, categorizing them into proestrus, estrus, metestrus, and diestrus (Nallathambi and Bhargavan 2019).

### Tissue sampling and processing

By the end of the experiment, overnight fasting rats from all groups at diestrus stage were anesthetized by i.p pentobarbital sodium (50 mg/kg), blood was collected by intracardiac puncture, permitted to coagulate for 10 min at ambient temperature, followed by centrifugation for 20 min at a velocity of 3000 rpm to procure serum for the assessment of serum concentrations of testosterone, follicle-stimulating hormone (FSH), LH, fasting insulin, and glucose for the evaluation of IR via HOMA-IR. The abdomen was opened. Both ovaries were isolated and washed with cold saline; then, OW was measured. The right ovary was preserved in 10% formalin for histological investigation under light microscopy, while the left ovary was homogenized in 7 ml of phosphate-buffered saline (PBS). The homogenates were centrifuged for 20 min at 3000 rpm at 4 °C; the supernatant was collected and stored at − 80 °C until the tissue parameters SIRT1 and TNFα were analyzed.

## Biochemical analysis

### Assessment of testosterone hormone level in serum

Serum testosterone hormone level was measured by ELISA using a kit obtained from Sun Red Biotechnology Company, Shanghai (catalog no. 201–11-5126).

#### Determination of FSH/LH ratio

For FSH, the kit is a competitive inhibition enzyme-linked immunosorbent assay (ELISA) supplied by DLDEVELOP, Canada (catalog no. DL-FSH-Ra).

For LH, the kit is a competitive inhibition ELISA supplied by DLDEVELOP, Canada (catalog no. DL-LH-Ra).

### Assessment of IR (HOMA-IR)

For calculation of (HOMA-IR) = (fasting glucose (mmol/l) × fasting insulin (mIU/L)/22.5 (Chen et al. 2021).

The insulin kit is a competitive inhibition ELISA supplied by DLDEVELOP, Canada (catalog no. DL-INS-Ra).

For glucose, serum glucose level was determined using the enzymatic colorimetric method supplied by Biodiagnostic.

#### Determination of TNFα in tissue

The kit is a sandwich ELISA supplied by DLDEVELOP, Canada (catalog no. DL-TNFα-Ra).

#### Determination of SIRT1 in tissue

The kit is a sandwich ELISA supplied by DLDEVELOP, Canada (catalog no. DL-SIRT1-Ra).

### Histopathological examination

The right ovary was promptly preserved in 10% formaldehyde. Paraffin slices (5 µm) were prepared and stained with H&E; the slides were analyzed under a light microscope for histological alterations. Granulosa and theca cell layers, together with several follicle types (primordial, primary (PF), secondary (SF), tertiary (TF), luteinized (LF), Graafian (GF), cystic (CF), atretic (AF), and corpus luteum (CL)), were detected across different groups.

### Statistical analysis

All collected data were organized into tables and subjected to statistical analysis utilizing GraphPad Prism version 5 for Windows (2007, GraphPad Software, Inc.). The Shapiro–Wilk test for normality was conducted. The parametric values were expressed as mean ± standard deviation (SD) and analyzed using one-way ANOVA, post hoc Tukey’s multiple comparison tests, and paired-samples t-test to identify differences between beginning and final BW. The parametric values’ correlation coefficient (*r*) is referred to as Pearson’s correlation coefficient. The *F* value is computed, and the *p*-value is established, with significance regarded at *p* < 0.05.

## Results

### Effect of different treatments on BW (gm) and OW (mg) within each one of the studied groups

**Fig. 2:**
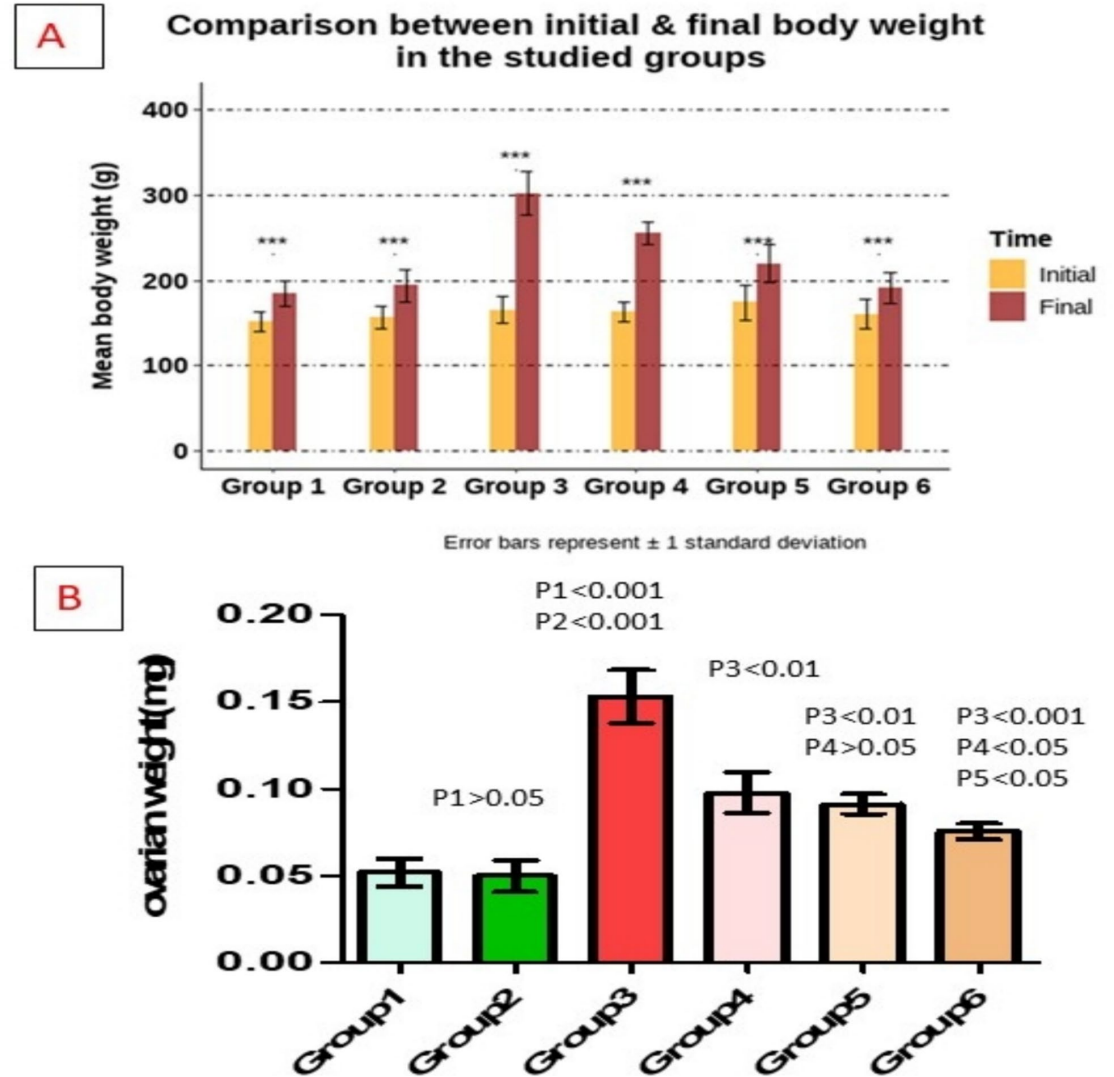
**A** Difference between the initial and final body weight within each studied group. **B** Changes in ovarian weight. P1: difference compared to group 1, P2: difference compared to group 2, P3: difference compared to group 3, P4: difference compared to group 4, P5: difference compared to group 5. Group 1: control group, Group 2: vehicle group, Group 3: untreated PCOS group, Group 4: metformin-treated group, Group 5: bromelain-treated group, Group 6: combined group

BW was significantly increase in untreated-PCOS group compared to (control and vehicle) groups, significantly decreased in (metformin-treated, bromelain-treated, and combined) groups compared to untreated-PCOS group also decreased in (bromelain-treated and combined) compared to metformin-treated group and decreased in combined group compared to bromelain-treated group (*p* < 0.05) (Table 1, Fig. 2A). OW was significantly increase in untreated-PCOS group compared to (control and vehicle) groups, significantly decreased in (metformin-treated, bromelain-treated and combined) groups compared to untreated-PCOS group also decreased in combined compared to (metformin-treated and bromelain-treated) groups (*p* < 0.05) (Table 1, Fig. 2B).

**Table 1:** Percent change in BW and OW in different studied groups

Parameter	*n*	Control	Vehicle	PCOS	PCOS + metformin	PCOS + bromelain	Combined	*p*-value
Percent change in BW	8	21.63 ± 2.568	23.40 ± 2.115	65.48 ± 5.967*^,#^	41.53 ± 4.191^$^	34.80 ± 5.029^$,@^	27.60 ± 3.373^$,@,&^	*p* < 0.001
		MD1 (95%CI)	− 2.4 (− 12.4–7.6)	− 60.9 (− 70.9– − 51)	− 37.7 (− 47.7– − 27.8)	− 24.2 (− 34.2– − 14.2)	− 6.4 (− 16.4–3.53)	
		MD2 (95%CI)		− 58.6 (− 68.58– − 48.63)	− 35.34 (− 45.32– − 25.37)	− 21.81 (− 31.78– − 11.84)	− 4.06 (− 14.03–5.91)	
		MD3 (95%CI)			23.26 (13.29–33.23)	36.79 (26.82–46.77)	54.55 (44.57–64.52)	
		MD4 (95%CI)				13.53 (3.56–23.51)	31.28 (21.31–41.26)	
		MD5 (95%CI)					17.75 (7.78–27.72)	
OW (mg)	8	52 ± 21.5	49.9 ± 23.9	153 ± 40^*,#^	97.8 ± 31^$^	91 ± 15^$^	76 ± 12^$,@,&^	*p* < 0.001
		MD1 (95%CI)	0 (− 0.04–0.03)	− 0.11 (− 0.14– − 0.07)	− 0.05 (− 0.09– − 0.01)	− 0.04 (− 0.07–0)	− 0.02 (− 0.06–0.01)	
		MD2 (95%CI)		− 0.1 (− 0.14– − 0.07)	− 0.05 (− 0.08– − 0.01)	− 0.04 (− 0.07–0)	− 0.02 (− 0.06–0.01)	
		MD3 (95%CI)			0.06 (0.02–0.1)	0.07 (0.03–0.11)	0.08 (0.04–0.12)	
		MD4 (95%CI)				0.01 (− 0.03–0.05)	0.02 (− 0.01–0.06)	
		MD5 (95%CI)					0.01 (− 0.02–0.05)	

Data presented as mean ± SD. *BW*, body weight; *OW*, ovarian weight; *PCOS*, polycystic ovary syndrome. ^*^Significant compared to the control group. ^#^Significant compared to the vehicle group. ^$^Significant compared to the untreated-PCOS group. ^@^Significant compared to metformin-treated-PCOS group. ^&^Significant compared to bromelain-treated-PCOS group

### Effect of different treatments on serum hormonal levels of testosterone, FSH, and LH (FSH/LH ratio) within each of the studied groups

**Fig. 3:**
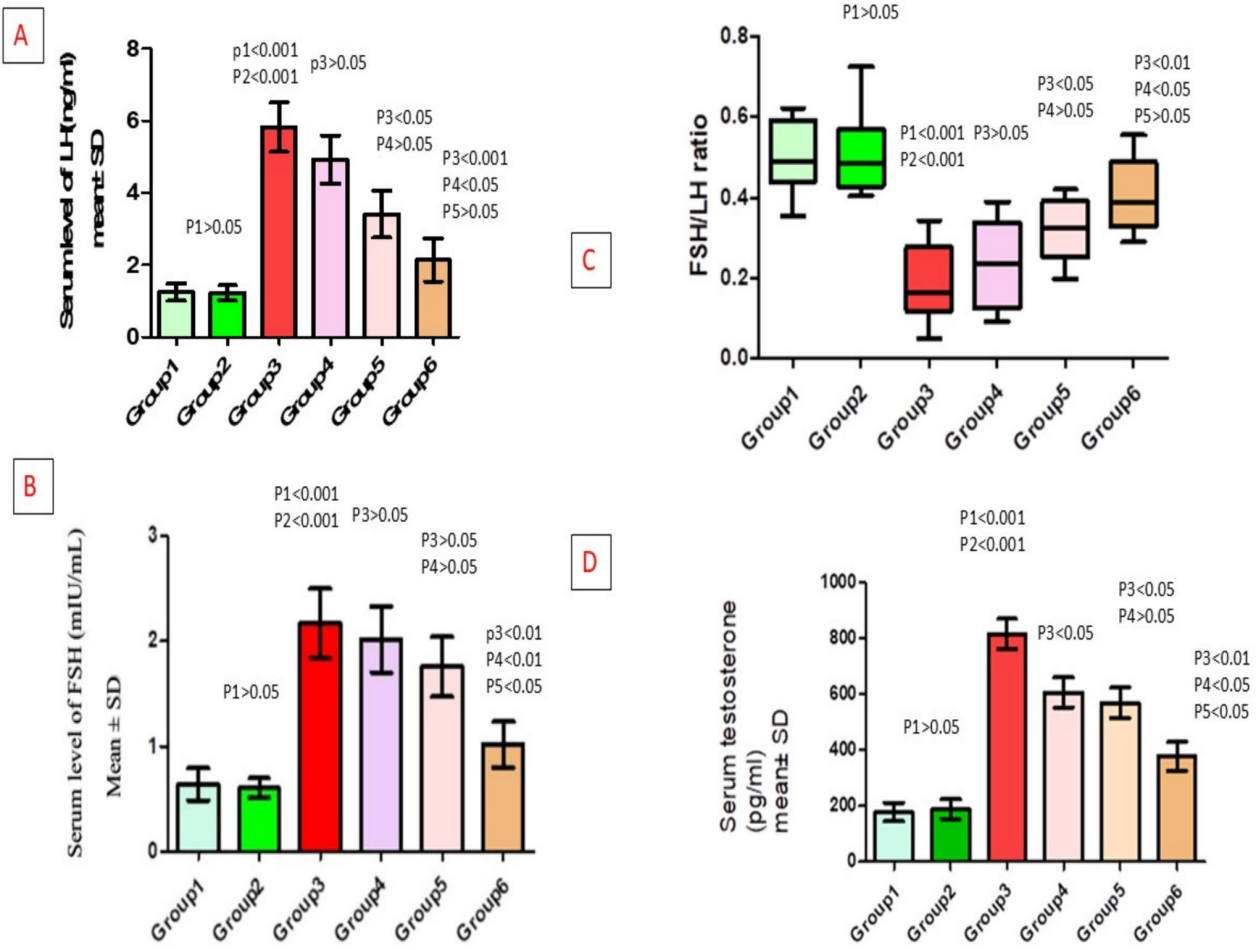
**A** Changes in serum levels of LH in different studied groups. **B** Changes in serum levels of FSH. **C** Changes in FSH/LH ratio. **D** Changes in serum levels of testosterone. P1: difference compared to group 1, P2: difference compared to group 2, P3: difference compared to group 3, P4: difference compared to group 4, P5: difference compared to group 5. Group 1: control group, Group 2: vehicle group, Group 3: untreated PCOS group, Group 4: metformin-treated group, Group 5: bromelain-treated group, Group 6: combined group

Serum testosterone level was significantly increased in the untreated-PCOS group compared to the (control and vehicle) groups, significantly decreased in (metformin-treated, bromelain-treated and combined) groups compared to the untreated-PCOS group, and also decreased in combined compared to (metformin-treated and bromelain-treated) groups (*p* < 0.05) (Table 2, Fig. 3D).

**Table 2:** Hormonal levels in different studied groups

Parameter	***n***	Control	Vehicle	PCOS	PCOS + metformin	PCOS + bromelain	Combined	***p***-value
Serum testosterone level (pg/ml)	8	179.4 ± 94.59	189 ± 100.3	815.2 ± 153.9^*,#^	606.1 ± 151.1^$^	569.9 ± 153.9^$^	377.7 ± 143.6^$,@,&^	*p* < 0.001
		MD1 (95%CI)	− 9.63 (− 211.56–192.31)	− 635.8 (− 837.73– − 433.87)	− 426.71 (− 628.65– − 224.78)	− 390.56 (− 592.5– − 188.63)	− 198.36 (− 400.3–3.57)	
		MD2 (95%CI)		− 626.18 (− 828.11– − 424.24)	− 417.09 (− 619.02– − 215.15)	− 380.94 (− 582.87– − 179)	− 188.74 (− 390.67–13.2)	
		MD3 (95%CI)			209.09 (7.15–411.02)	245.24 (43.3–447.17)	437.44 (235.5–639.37)	
		MD4 (95%CI)				36.15 (− 165.78–238.08)	228.35 (26.42–430.28)	
		MD5 (95%CI)					192.2 (− 9.73–394.13)	
Serum LH level (ng/ml)	8	1.246 ± 0.681	1.220 ± 0.595	5.821 ± 1.938^*,#^	4.921 ± 1.9	3.410 ± 1.842^$^	2.143 ± 1.685^$,@^	*p* < 0.01
		MD1 (95%CI)	0.04 (− 0.89–0.96)	− 1.53 (− 2.45– − 0.6)	− 0.39 (− 1.31–0.54)	− 0.55 (− 1.48–0.38)	− 0.38 (− 1.3–0.55)	
		MD2 (95%CI)		− 1.57 (− 2.49– − 0.64)	− 0.42 (− 1.35–0.5)	− 0.59 (− 1.51–0.34)	− 0.42 (− 1.34–0.51)	
		MD3 (95%CI)			1.14 (0.22–2.07)	0.98 (0.05–1.9)	1.15 (0.23–2.08)	
		MD4 (95%CI)				− 0.16 (− 1.09–0.76)	0.01 (− 0.92–0.93)	
		MD5 (95%CI)					0.17 (− 0.75–1.1)	
Serum FSH level (IU/ml)	8	0.639 ± 0.331	0.601 ± 0.266	0.181 ± 0.099^*,#^	0.24 ± 0.111	0.32 ± 0.078	0.405 ± 0.092^$,@,&^	*p* < 0.01
		MD1 (95%CI)	0.03 (− 2.29–2.34)	− 4.57 (− 6.89– − 2.26)	− 3.67 (− 5.99– − 1.36)	− 2.16 (− 4.48–0.15)	− 0.89 (− 3.2–1.43)	
		MD2 (95%CI)		− 4.6 (− 6.91– − 2.29)	− 3.7 (− 6.01– − 1.39)	− 2.19 (− 4.5–0.12)	− 0.91 (− 3.23–1.4)	
		MD3 (95%CI)			0.9 (− 1.41–3.21)	2.41 (0.1–4.72)	3.69 (1.37–6)	
		MD4 (95%CI)				1.51 (− 0.8–3.82)	2.79 (0.47–5.1)	
		MD5 (95%CI)					1.28 (− 1.04–3.59)	
FSH/LH ratio	8	0.498 ± 0.089	0.509 ± 0.0105	0.362 ± 0.071^*,#^	0.4 ± 0.099	0.485 ± 0.079	0.545 ± 0.096^$,@^	*p* < 0.01
		MD1 (95%CI)	− 0.01 (− 0.15–0.13)	0.32 (0.17–0.46)	0.26 (0.11–0.4)	0.18 (0.03–0.32)	0.09 (− 0.05–0.24)	
		MD2 (95%CI)		0.33 (0.18–0.47)	0.27 (0.13–0.41)	0.19 (0.04–0.33)	0.1 (− 0.04–0.25)	
		MD3 (95%CI)			− 0.06 (− 0.2–0.08)	− 0.14 (− 0.28–0)	− 0.22 (− 0.37– − 0.08)	
		MD4 (95%CI)				− 0.08 (− 0.22–0.06)	− 0.17 (− 0.31– − 0.02)	
		MD5 (95%CI)					− 0.08 (− 0.23–0.06)	

Data presented as mean ± SD. ^*^Significant compared to the control group. ^#^Significant compared to the vehicle group. ^$^Significant compared to the untreated-PCOS group. ^@^Significant compared to metformin-treated-PCOS group. ^&^Significant compared to bromelain-treated-PCOS group

Serum LH level was significantly increased in untreated-PCOS group compared to (control and vehicle) groups, significantly decreased in (bromelain-treated and combined) groups compared to untreated-PCOS group, and also significantly decreased in combined compared to metformin-treated group (*p* < 0.05) (Table 2, Fig. 3A). Serum FSH level was significantly increased in untreated-PCOS group compared to (control and vehicle) groups, significantly decreased in combined group compared to (metformin-treated and bromelain-treated) groups (*p* < 0.01, *p* < 0.05, respectively) (Table 2, Fig. 3B). Serum FSH/LH ratio was significantly decrease in untreated-PCOS group compared to (control and vehicle) groups and significantly increased in combined group compared to metformin-treated group (*p* < 0.05) (Table 2, Fig. 3C).

### Effect of different treatments on fasting serum insulin and glucose (HOMA-IR) within each one of the studied groups

**Fig. 4:**
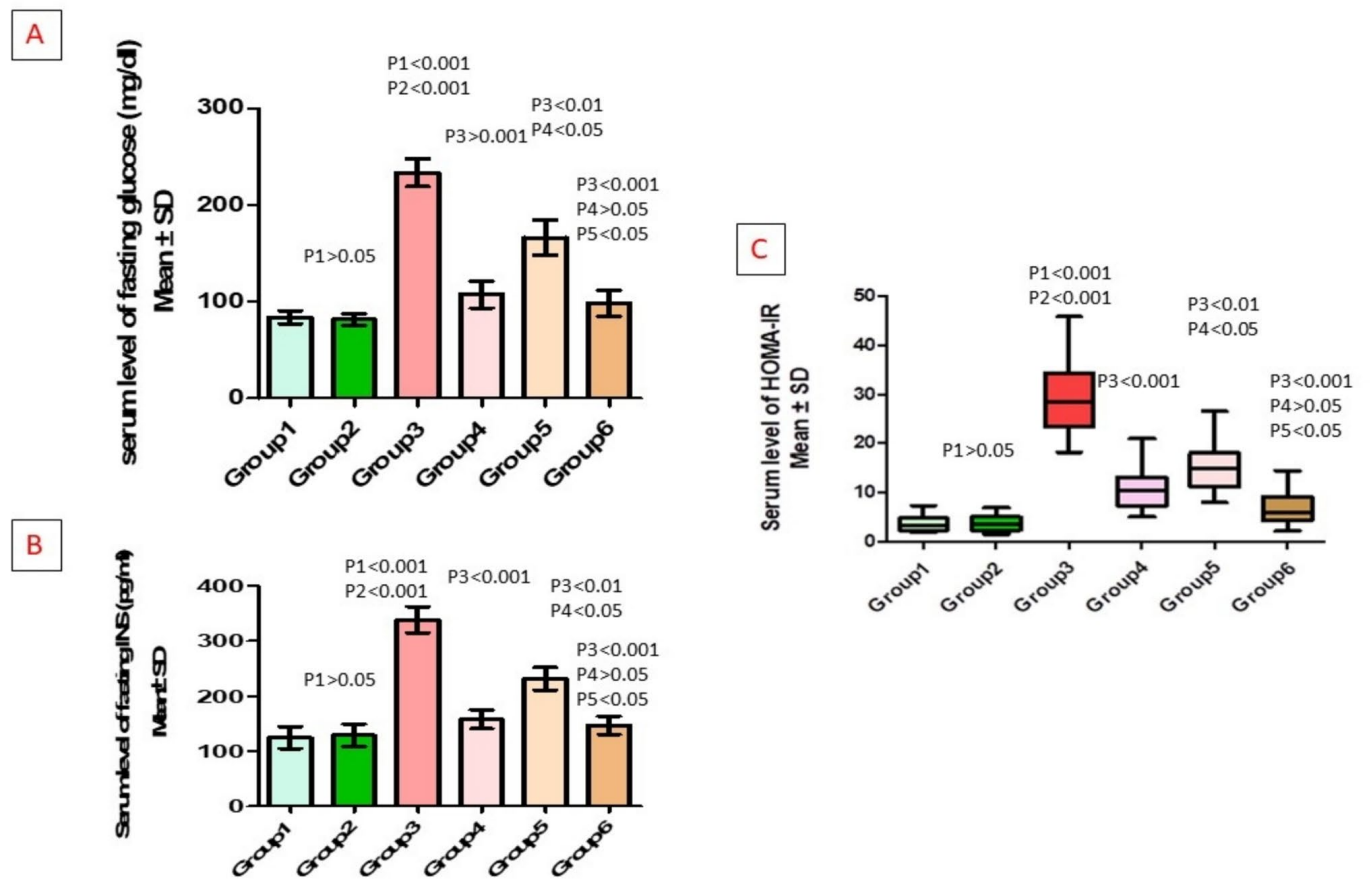
**A** Changes in serum levels of fasting glucose in different studied groups. **B** Changes in fasting insulin (INS). **C** Changes in levels of HOMA-IR. P1: difference compared to group 1, P2: difference compared to group 2, P3: difference compared to group 3, P4: difference compared to group 4, P5: difference compared to group 5. Group 1: control group, Group 2: vehicle group, Group 3: untreated PCOS group, Group 4: metformin-treated group, Group 5: bromelain-treated group, Group 6: combined group

Serum fasting glucose, fasting insulin, and HOMA-IR were significantly increased in the untreated-PCOS group compared to the control and vehicle groups. Compared to the untreated-PCOS group, they were significantly decreased in the metformin-treated, bromelain-treated, and combined treatment groups. Additionally, levels were significantly lower in the metformin-treated group compared to the bromelain-treated group (*p* < 0.05) and decreased in the combined group compared to the bromelain-treated group (*p* < 0.05) (Table 3, Fig. 4).

**Table 3:** HOMA-IR in different studied groups

Parameter	*n*	Control	Vehicle	PCOS	PCOS + metformin	PCOS + bromelain	Combined	*p*-value
Serum fasting glucose (mg/dl)	8	83.91 ± 19.30	81.59 ± 18.11	233.6 ± 40.33^*,#^	107.5 ± 39.55^$^	166.3 ± 51.25^$,@^	98.31 ± 39.06^$,&^	*p* < 0.001
		MD1 (95%CI)	2.33 (− 52.33–56.98)	− 149.71 (− 204.36– − 95.06)	− 23.59 (− 78.24–31.06)	− 82.34 (− 136.99– − 27.69)	− 14.4 (− 69.05–40.25)	
		MD2 (95%CI)		− 152.04 (− 206.69– − 97.39)	− 25.91 (− 80.56–28.74)	− 84.66 (− 139.31– − 30.01)	− 16.73 (− 71.38–37.93)	
		MD3 (95%CI)			126.13 (71.47–180.78)	67.38 (12.72–122.03)	135.31 (80.66–189.96)	
		MD4 (95%CI)				− 58.75 (− 113.4– − 4.1)	9.19 (− 45.46–63.84)	
		MD5 (95%CI)					67.94 (13.29–122.59)	
Serum fasting insulin (pg/dl)	8	124.8 ± 55.9	129.2 ± 58.69	338.4 ± 67.88^*,#^	207.2 ± 48.69^$^	231.9 ± 59.80^$,@^	183.7 ± 46.21^$,&^	*p* < 0.001
		MD1 (95%CI)	− 4.34 (− 88.9–80.23)	− 213.54 (− 298.1– − 128.97)	− 33.3 (− 117.87–51.27)	− 106.93 (− 191.49– − 22.36)	− 22.68 (− 107.24–61.89)	
		MD2 (95%CI)		− 209.2 (− 293.77– − 124.63)	− 28.96 (− 113.53–55.6)	− 102.59 (− 187.15– − 18.02)	− 18.34 (− 102.9–66.23)	
		MD3 (95%CI)			180.24 (95.67–264.8)	106.61 (22.05–191.18)	190.86 (106.3–275.43)	
		MD4 (95%CI)				− 73.63 (− 158.19–10.94)	10.63 (− 73.94–95.19)	
		MD5 (95%CI)					84.25 (− 0.32–168.82)	
HOMA-IR	8	3.791 ± 1.778	3.819 ± 1.735	29.68 ± 8.423^*,#^	11 ± 4.923^$^	19.37 ± 8.423^$,@^	6.885 ± 3.883^$,&^	*p* < 0.001
		MD1 (95%CI)	− 0.03 (− 7.46–7.41)	− 25.89 (− 33.32– − 18.45)	− 7.21 (− 14.64–0.22)	− 11.58 (− 19.01– − 4.15)	− 3.09 (− 10.53–4.34)	
		MD2 (95%CI)		− 25.86 (− 33.29– − 18.43)	− 7.18 (− 14.62–0.25)	− 11.55 (− 18.99– − 4.12)	− 3.07 (− 10.5–4.37)	
		MD3 (95%CI)			18.68 (11.24–26.11)	14.31 (6.87–21.74)	22.79 (15.36–30.23)	
		MD4 (95%CI)				− 4.37 (− 11.8–3.06)	4.12 (− 3.31–11.55)	
		MD5 (95%CI)					8.49 (1.05–15.92)	

Data presented as mean ± SD. ^*^Significant compared to the control group. ^#^Significant compared to the vehicle group. ^$^Significant compared to the untreated-PCOS group. ^@^Significant compared to metformin-treated-PCOS group. ^&^Significant compared to bromelain-treated-PCOS group

### Effect of different treatments on inflammatory marker (TNFα) within each one of the studied groups

**Fig. 5:**
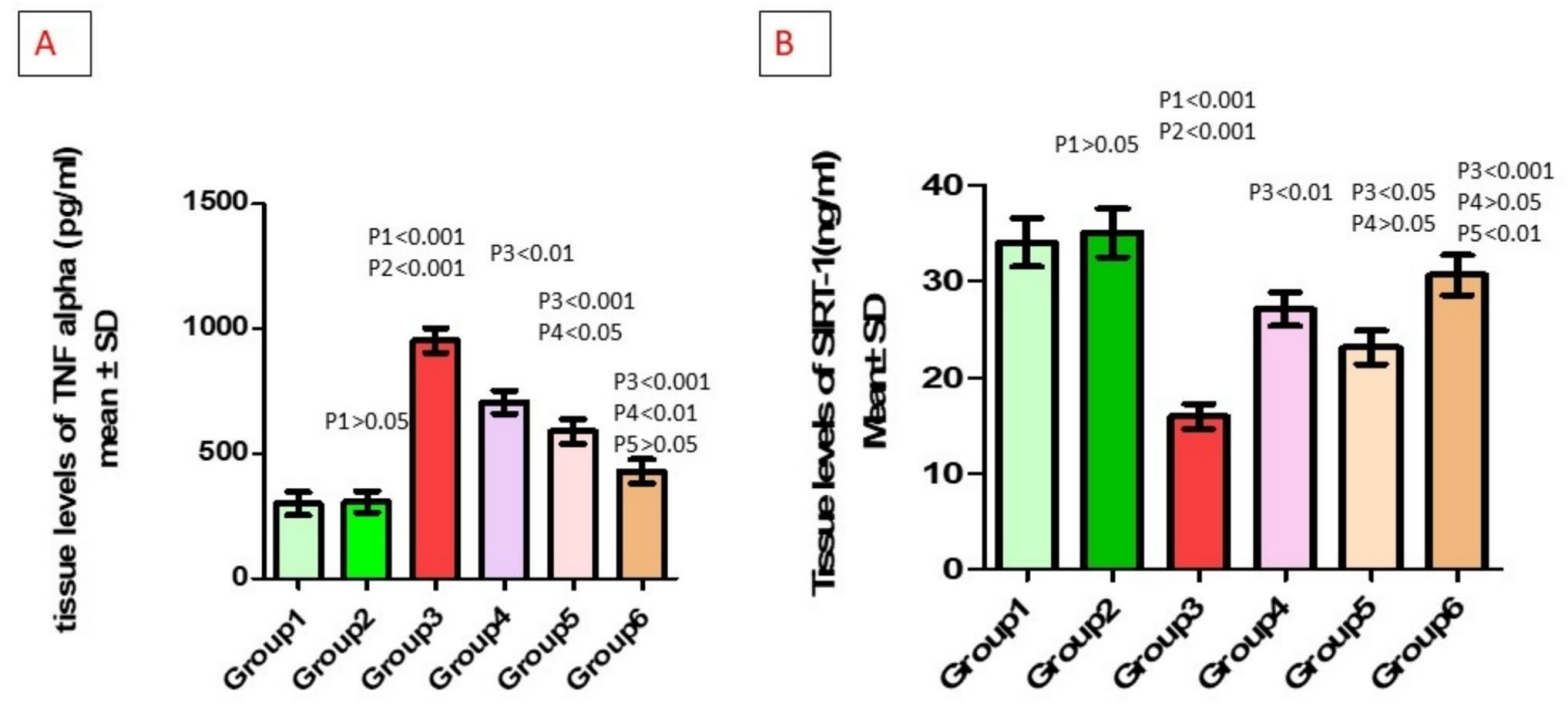
**A** Changes in tissue levels of TNFα in different studied groups. **B** Changes in tissue levels of SIRT1. P1: difference compared to group 1, P2: difference compared to group 2, P3: difference compared to group 3, P4: difference compared to group 4, P5: difference compared to group 5. Group 1: control group, Group 2: vehicle group, Group 3: untreated PCOS group, Group 4: metformin-treated group, Group 5: bromelain-treated group, Group 6: combined group

Tissue levels of TNFα were significantly increased in the untreated-PCOS group compared to the control and vehicle groups. Compared to the untreated-PCOS group, they were significantly decreased in the metformin-treated, bromelain-treated, and combined treatment groups. Additionally, TNFα levels were significantly lower in the bromelain-treated and combined groups compared to the metformin-treated group (*p* < 0.05, *p* < 0.01, respectively) (Table 4, Fig. 5A).

**Table 4:** Tissue levels of TNFα and SIRT1 in different studied groups

Parameter	*n*	Control	Vehicle	PCOS	PCOS + metformin	PCOS + bromelain	Combined	*p*-value
Tissue level of TNFα (pg/ml)	8	301.5 ± 131.9	308.4 ± 122.3	953.5 ± 141^*,#^	706.8 ± 130.3^$^	589.8 ± 140.5^$,@^	430.6 ± 134.6^$,@^	*p* < 0.001
		MD1 (95%CI)	− 6.94 (− 206.32–192.44)	− 651.95 (− 851.33– − 452.57)	− 405.3 (− 604.68– − 205.92)	− 288.25 (− 487.63– − 88.87)	− 129.13 (− 328.5–70.25)	
		MD2 (95%CI)		− 645.01 (− 844.39– − 445.63)	− 398.36 (− 597.74– − 198.98)	− 281.31 (− 480.69– − 81.93)	− 122.19 (− 321.57–77.19)	
		MD3 (95%CI)			246.65 (47.27–446.03)	363.7 (164.32–563.08)	522.83 (323.45–722.2)	
		MD4 (95%CI)				117.05 (− 82.33–316.43)	276.18 (76.8–475.55)	
		MD5 (95%CI)					159.13 (− 40.25–358.5)	
Tissue level of SIRT1 (ng/ml)	8	34.06 ± 7.131	35.09 ± 7.179	15.95 ± 3.678^*,#^	29.14 ± 4.988^$^	25.56 ± 5.567^$^	32.67 ± 5.872^$,&^	*p* < 0.01
		MD1 (95%CI)	− 1.03 (− 9.65–7.59)	18.11 (9.49–26.73)	6.92 (− 1.7–15.54)	10.9 (2.28–19.52)	3.39 (− 5.23–12.01)	
		MD2 (95%CI)		19.14 (10.52–27.76)	7.95 (− 0.67–16.57)	11.93 (3.31–20.55)	4.42 (− 4.2–13.04)	
		MD3 (95%CI)			− 11.19 (− 19.81– − 2.57)	− 7.21 (− 15.83–1.41)	− 14.72 (− 23.34– − 6.1)	
		MD4 (95%CI)				3.98 (− 4.64–12.6)	− 3.53 (− 12.15–5.09)	
		MD5 (95%CI)					− 7.51 (− 16.13–1.11)	

Data was presented as mean ± SD. ^*^Significant compared to the control group. ^#^Significant compared to the vehicle group. ^$^Significant compared to the untreated-PCOS group. ^@^Significant compared to metformin-treated-PCOS group. ^&^Significant compared to bromelain-treated-PCOS group

### Effect of different treatments on SIRT1within each one of the studied groups

The tissue level of SIRT1 was significantly decreased in the untreated-PCOS group compared to the control and vehicle groups. It was significantly increased in the metformin-treated, bromelain-treated, and combined treatment groups compared to the untreated-PCOS group. Additionally, SIRT1 levels were significantly higher in the combined group compared to the bromelain-treated group (*p* < 0.01) (Table 4, Fig. 5B).

### Changes in vaginal smear in different studied groups

**Fig. 6:**
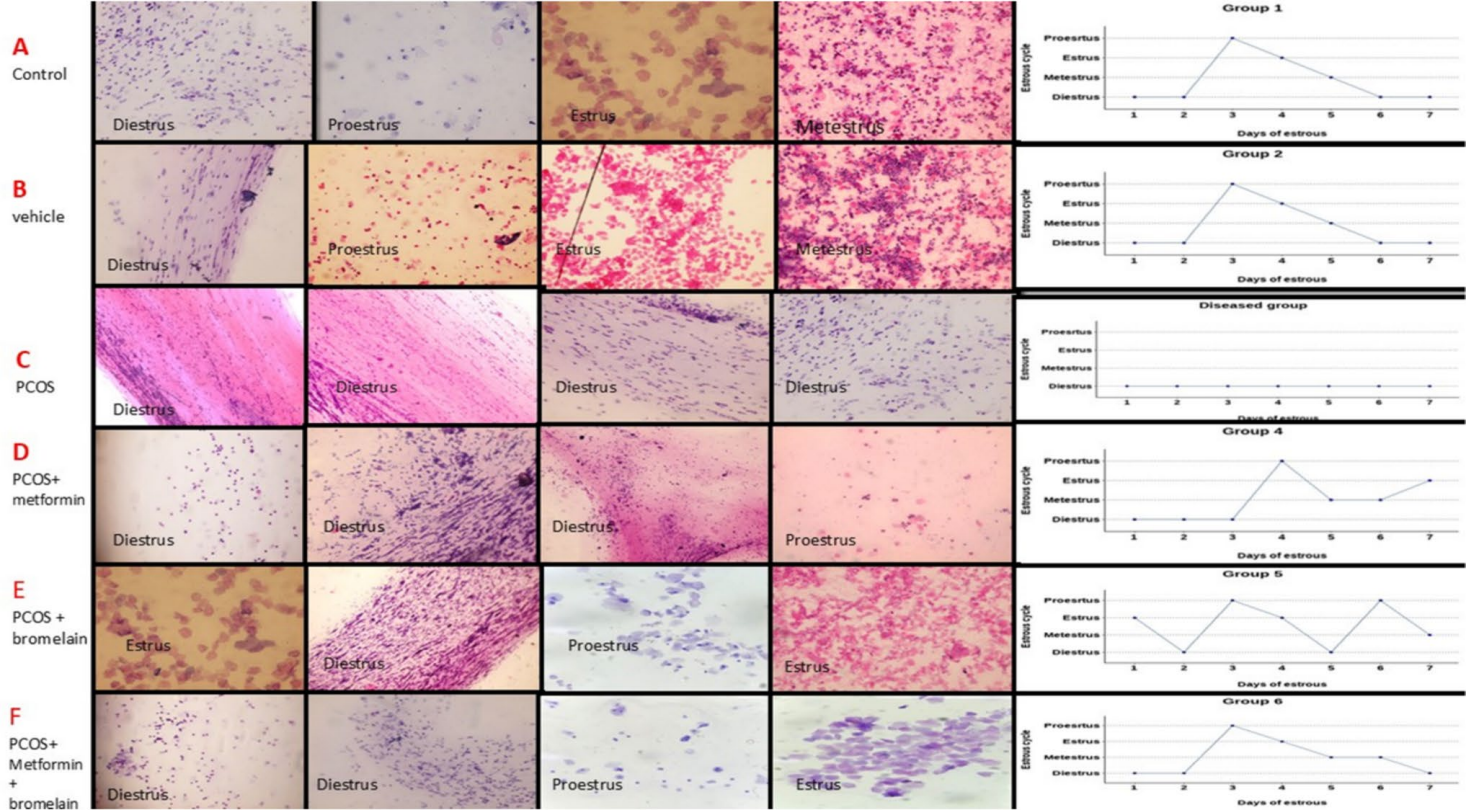
Assessment of vaginal smear in different studied groups

**Fig. 7:**
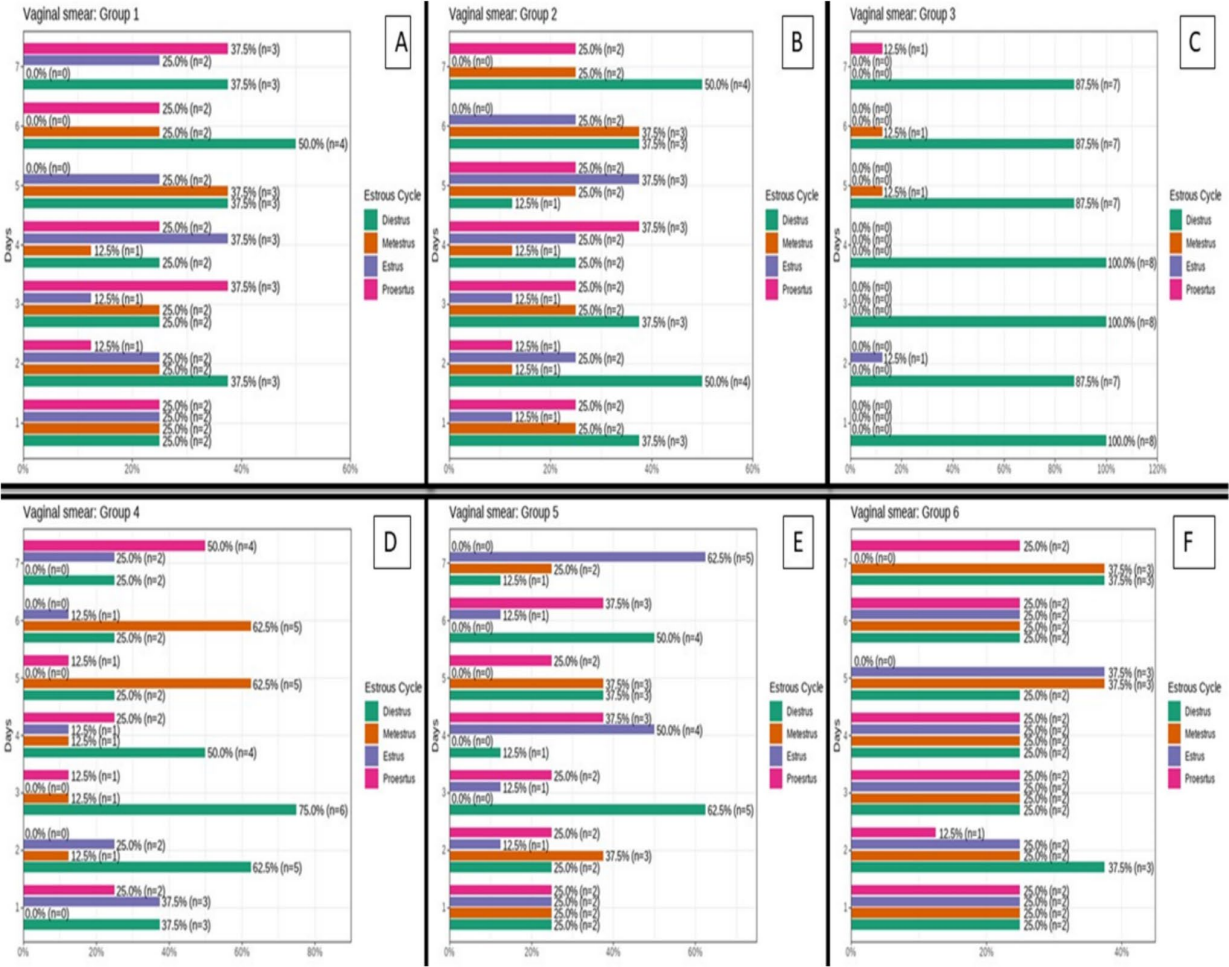
Percent of different stages along seven days vaginal smear and number of rats representing different stages

Control group showed normal estrous cycle with average 7 days started with diestrus stage for 2 days which is characterized by massive infiltration of neutrophils with low number of nucleated and a nucleated keratinized epithelial cells, followed by proestrus stage for 1 day which is characterized by presence of rounded nucleated epithelial cells and keratinized a nucleated cells, followed by estrus stage for one day which is characterized by presence of nucleated keratinized epithelial cells, followed by metestrus stage for 1 day which is characterized by beginning of emergence of neutrophils among a nucleated epithelial cells and followed by diestrus stage again for 2 days (Figs. 6A and 7A). The vehicle group showed a normal estrous cycle, as did the control group (Figs. 6B and 7B). The untreated PCOS group showed arrest of the estrus cycle at the diestrus stage on all days, which is characterized by massive infiltration of neutrophils with a low number of nucleated and a-nucleated keratinized epithelial cells (Figs. 6C and 7C). The metformin-treated group revealed improvement in the estrus cycle compared to the untreated PCOS group but with skipped stages in between and longer duration of the stage (Figs. 6D and 7D). Bromelain-treated group showed better improvement in estrus cycle compared to the untreated PCOS group; stages of cycle were normal in duration but irregular (Figs. 6E and 7E). The combined group exhibited better improvement in the estrus cycle than the untreated PCOS group, whose cycle stages were regular and had an average duration (Figs. 6F and 7F).

### Light microscope histopathological examination of the ovarian sections detected by H&E stain

**Fig. 8:**
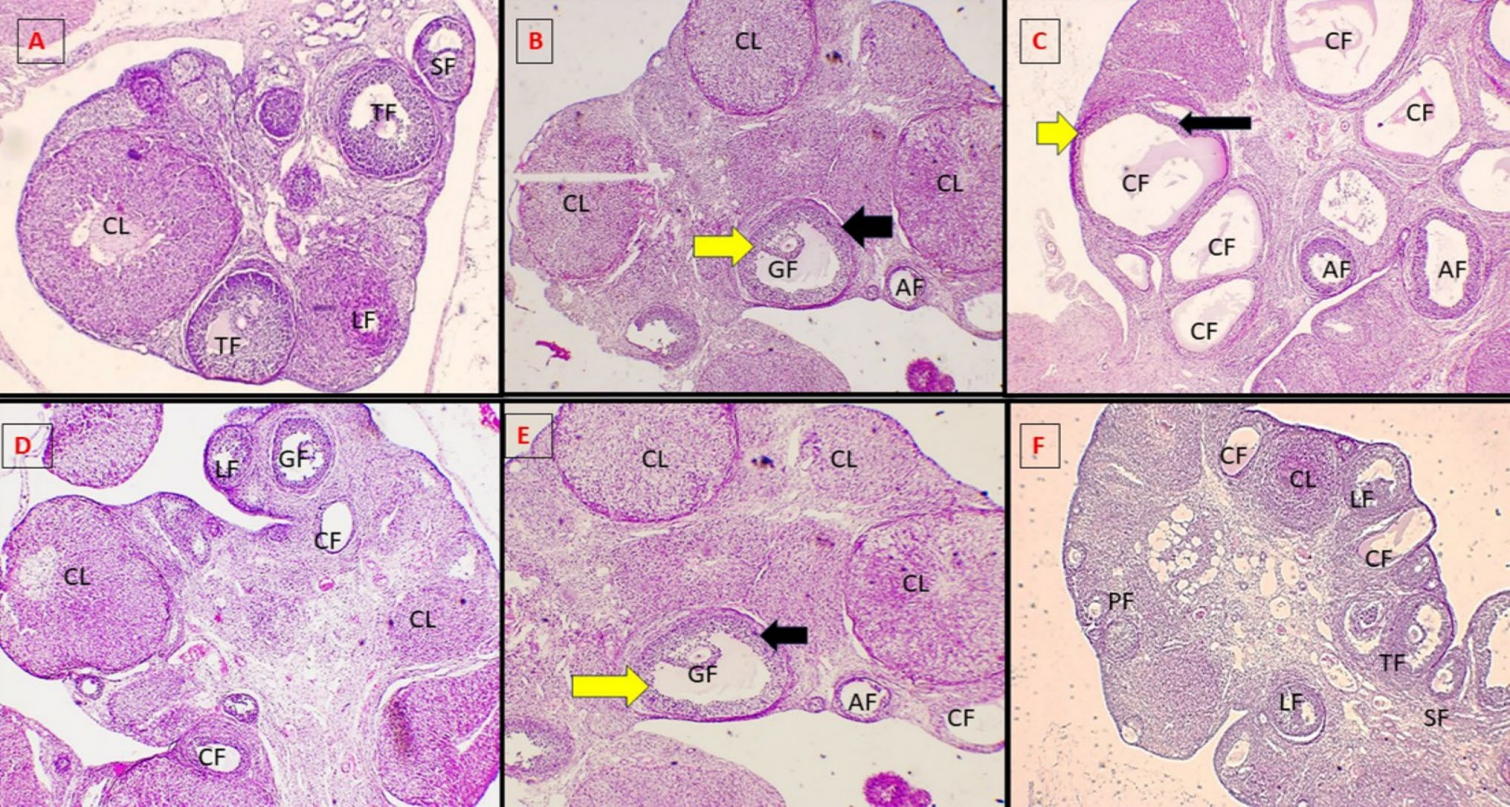
Histopathological picture of H&E-stained ovarian section of different studied groups

Control group showed normal ovarian architecture and thickness with multiple follicles (SF, TF, LF) and CL (evidence of ovulation) (× 40) (Fig. 8A), also vehicle group showed normal ovarian architecture and thickness with mature GF with outer thin theca cell layer (black arrow) and inner thick layers of granulosa cells (yellow arrow), AF and CL (× 40) (Fig. 8B).

The untreated PCOS group showed disturbed ovarian architecture and thickness with multiple variable-sized CF and AF with absence of mature Graafian follicles and corpora (× 40), CF with bands of luteinized theca cells (black arrow) and inner thin layer of granulosa cells (yellow arrow) (× 200) (Fig. 8C).

Metformin treated group showed the appearance of mature GF, LF, and multiple corpora with some cysts (× 40) (Fig. 8D), also bromelain treated group showed the appearance of mature GF with outer thin theca cell layer (black arrow) and inner thick layers of granulosa cells (yellow arrow) and multiple corpora with some atretic and CF (× 40) (Fig. 8E), and combined group showed the appearance of multiple follicles (PF, SF, TF, and LF and multiple corpora) (× 40) (Fig. 8F).

## Discussion

The letrozole-induced PCOS model provides an ethically appropriate and technically feasible approach for investigating PCOS pathophysiology, overcoming the limitations inherent in human experimentation (Cui et al. 2023). This rodent model effectively recapitulates the key features of human PCOS, including hyperandrogenism, hormonal alterations, ovarian structural changes characterized by multi-cystic follicles, and metabolic dysfunction (Azhar et al. 2021). The observed hormonal alterations in letrozole-treated rats demonstrated a hyper-androgenized state consistent with the drug’s mechanism of preventing androgen-to-estrogen conversion through aromatase inhibition (Dumitrescu et al. 2015; Ibrahim et al. 2022). This increased intraovarian androgen production leads to follicular cyst formation with fluid accumulation and dysregulation of the hypothalamic-pituitary–gonadal axis, resulting in elevated serum LH levels (Tamadon et al. 2018) and FSH levels (Salehi et al. 2020) with a reduced FSH/LH ratio (Rajaei et al. 2019; Zhou et al. 2021).

**Fig. 9:**
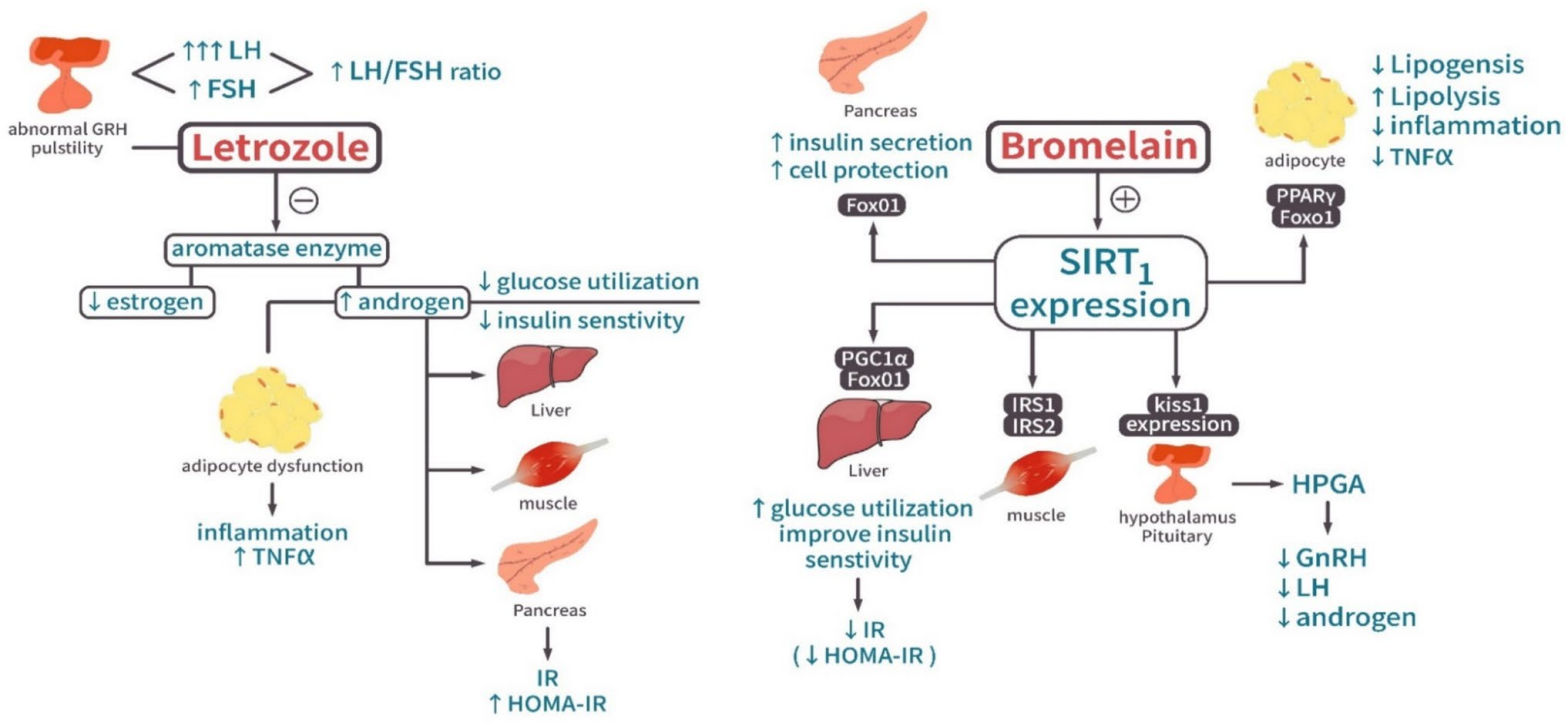
A mechanistic figure showed the mechanism of letrozole induction of PCOS and SIRT1 in controlling endocrine-metabolic disturbances

The study identified SIRT1 as a central mediator linking endocrine-metabolic disturbances in PCOS pathophysiology. Untreated PCOS animals exhibited significantly decreased SIRT1 expression, confirming that hyperandrogenemia disrupts the SIRT1 pathway and contributes to metabolic disorders (Jamshidi et al. 2021; Mihanfar et al. 2021). SIRT1’s protective function operates through multiple mechanisms, including deacetylation of FoxO1 to regulate autophagy, oxidative stress, and mitochondrial function (Wang and Li 2023), modulation of PPAR-γ to control glucose and lipid metabolism (Li et al. 2023), and suppression of kiss1 neurons leading to decreased GnRH release (Vazquez et al. 2025) (Fig. 9).

FoxO1, fork head box protein O1; PPAR γ, peroxisome proliferator activated receptor gamma; PGC1α, peroxisome proliferator activated receptor gamma coactivator-1 alpha; IRS1, insulin receptor substrate; Kiss1, kisspeptin receptor.

The interconnected nature of IR, inflammation, and weight gain was demonstrated through elevated HOMA-IR values and increased TNFα levels in untreated animals. Letrozole induction created a pathological cascade where adiposity, mitochondrial dysfunction, and excess androgens contributed to pancreatic islet dysfunction, resulting in IR and subsequent weight gain (Gilbert et al. 2018; Zheng et al. 2021). This metabolic dysfunction triggered inflammatory responses through macrophage infiltration and adipose tissue inflammation, creating a self-perpetuating cycle of metabolic and endocrine disruption (Areloegbe et al. 2022).

Both metformin and bromelain demonstrated significant therapeutic benefits, though through distinct mechanisms. Metformin treatment effectively reduced testosterone levels by inhibiting androgen-induced ovarian theca cell proliferation and preventing follicular atresia (Ahmed et al. 2023; Tsai et al. 2023). The drug required a longer treatment duration for optimal FSH effects (La Marca et al. 2000) and exerted inhibitory effects on FSH at specific concentration ranges between 2.5 and 5 ng/ml (Rice et al. 2013), leading to non-significant differences in FSH/LH ratios (De Leo et al. 2000). The drug enhanced insulin sensitivity and glucose utilization (Goel et al. 2022; Hasoon et al. 2022), leading to decreased BW (Guan et al. 2020; Hu et al. 2020; Ashour et al. 2021), and attenuated adipose tissue inflammation while increasing SIRT1 protein expression in ovarian tissue (Tao et al. 2021; Xiao et al. 2022).

Bromelain treatment revealed complementary therapeutic effects, significantly reducing testosterone levels through its aromatase enzyme content (Blakemore and Naftolin 2016). The compound demonstrated anti-inflammatory properties by inhibiting nuclear factor kappa-B and mitogen-activated protein kinase signaling pathways (Pothacharoen et al. 2021). Additionally, bromelain activated the SIRT1 pathway by reducing oxidative stress, positioning it as a valuable therapeutic agent capable of inhibiting oxidative stress, inflammation, and apoptosis through SIRT1/AMPK pathway activation (Didamoony et al. 2022).

The combination therapy demonstrated superior efficacy to individual treatments, significantly improving hormonal balance, metabolic parameters, and ovarian morphology. These findings were corroborated by improvements in estrus cycle regulation, with treated animals showing recovery from the diestrus arrest observed in untreated PCOS groups (Kamal et al. 2022), and histopathological changes including restoration of normal ovarian architecture with reduced cyst formation and theca cell layer thickening (Lohrasbi et al. 2022). The improvements included the appearance of mature Graafian follicles, increased corpora lutea, and reduced cystic follicle numbers (Morsi et al. 2022; Sadeq et al. 2024).

### Limitations

The current study had potential limitations; the results in the present study singled out the outcome of letrozole-induced PCOS in rats, and there were species-specific differences between humans and rats in reproductive physiology, metabolism, and hormonal regulation. Letrozole induction mimics only certain aspects but not all the syndrome features in humans, and small sample sizes limit generalizability. Rats have a 4–5-day estrous cycle, which is different from the human menstrual cycle, in addition to a limited study duration with a shorter life span of rats. The 2-week treatment period was sufficient to demonstrate significant amelioration of endocrine-metabolic parameters. However, the chronic effects and long-term safety remain unaddressed.

### Future directions

Bromelain demonstrates potential as an adjuvant therapy for PCOS through modulation of metabolic and inflammatory pathways, particularly via SIRT1 activation. Its synergistic effects with metformin support a multi-targeted approach to addressing the complex pathophysiology of PCOS. To facilitate clinical translation, several key challenges must be addressed. First, the development of improved delivery systems enhances the oral bioavailability of bromelain. Second, dose extrapolation from animal models requires careful validation to account for interspecies metabolic differences. Although bromelain is generally regarded as safe, its anticoagulant properties raise concerns regarding potential drug interactions.

Furthermore, the lack of long-term safety data underscores the need for comprehensive toxicological and reproductive studies. Future research should evaluate the chronic effects of bromelain, including its long-term therapeutic efficacy and potential side effects. Extended treatment durations (e.g., 4–6 weeks) should be employed to assess the sustainability of therapeutic outcomes, the risk of tachyphylaxis, and organ-specific safety parameters such as hepatic and renal function. Using transgenic animal models that more closely mimic human PCOS pathophysiology will further enhance the translational relevance of preclinical findings.

## Conclusion

The results of the present study signified that hormonal disturbance, IR, and inflammation are key players in the pathogenesis of PCOS and also explained the effective role of SIRT1. This study revealed the better effects of bromelain on endocrine-metabolic aspects of disease, and its role as anti-inflammatory also suggested that a combination of metformin and bromelain provided an additional therapeutic role and more improvement as regards their effect on reduction of BW, as well as their effective role on metabolic and hormonal levels, respectively. These encouraging results support using bromelain as an adjuvant therapy to metformin to cover all aspects of the disease’s pathophysiology. Considering limitations, these results should be verified in further animal and human clinical studies over a longer time.

## Supplementary Information

Below is the link to the electronic supplementary material.

Supplementary file (DOCX 37.2 KB)

## Data Availability

Supplementary data is available upon inquiry.

## Abbreviations

*PCOS*: Polycystic ovary syndrome
*SIRT1*: Silencing information regulator 2-related enzyme 1
*HOMA-IR*: Homeostasis model assessment of insulin resistance
*TNFα*: Tumor necrosis factor alpha
*LH*: Luteinizing hormone
*FSH*: Follicle-stimulating hormone
*IR*: Insulin resistance
*CMC*: Carboxymethylcellulose
*H&E*: Hematoxylin and eosin
*PBS*: Phosphate-buffered saline
*ELISA*: Enzyme-linked immunosorbent assay
*SD*: Standard deviation
*ANOVA*: Analysis of variance
*BW*: Body weight
*PF*: Primary follicle
*SF*: Secondary follicle
*TF*: Tertiary follicle
*LF*: Luteinized follicle
*GF*: Graafian follicle
*CF*: Cystic follicle
*AF*: Atretic follicle
*CL*: Corpus luteum
