# Supplementary material for: Ameliorating effects of bromelain with or without metformin on endocrine-metabolic disturbances in letrozole-induced polycystic ovary syndrome in female rats via targeting SIRT1, insulin resistance, and inflammatory axis
Source: Naunyn Schmiedebergs Arch Pharmacol. 2025 Aug 30;399(2):2465–80. doi: 10.1007/s00210-025-04517-w (PMC12901086; doi:10.1007/s00210-025-04517-w)
Supplement: Supplementary file 1 — Supplementary file (DOCX 37.2 KB) [file 210_2025_4517_MOESM1_ESM.docx]

- **Tab1;** Values of percent change in body weight in different groups;

| Body weight | control | vehicle | diseased | metformin | bromelain | combined |
| --- | --- | --- | --- | --- | --- | --- |
| R1 | 22.759 | 22.378 | 85.161 | 56.886 | 38.431 | 27.333 |
| R2 | 19.497 | 23.529 | 84.375 | 53.714 | 42.914 | 26.571 |
| R3 | 18.667 | 25.111 | 80.838 | 60.113 | 40.357 | 22.325 |
| R4 | 21.429 | 21.053 | 68.571 | 68.105 | 66.521 | 30.517 |
| R5 | 20.112 | 23.188 | 86.207 | 55.232 | 35.211 | 28.341 |
| R6 | 23.457 | 24.858 | 87.059 | 76.47 | 55.302 | 20.511 |
| R6 | 20.975 | 27.272 | 83.694 | 46.667 | 47.408 | 29.762 |
| R8 | 22.222 | 20.805 | 81.123 | 53.751 | 36.53 | 35.302 |

- **Tab2;** Values of ovarian weight in different groups;

| Ovarian weight | control | vehicle | diseased | metformin | bromelain | combined |
| --- | --- | --- | --- | --- | --- | --- |
| R1 | 0.078 | 0.063 | 0.192 | 0.0952 | 0.0941 | 0.0636 |
| R2 | 0.065 | 0.07 | 0.129 | 0.0893 | 0.0812 | 0.0826 |
| R3 | 0.049 | 0.055 | 0.118 | 0.081 | 0.079 | 0.0752 |
| R4 | 0.029 | 0.028 | 0.183 | 0.11 | 0.103 | 0.0592 |
| R5 | 0.069 | 0.079 | 0.15 | 0.0781 | 0.0695 | 0.0895 |
| R6 | 0.054 | 0.042 | 0.0985 | 0.0697 | 0.113 | 0.0697 |
| R7 | 0.019 | 0.012 | 0.202 | 0.161 | 0.0995 | 0.0887 |
| R8 | 0.035 | 0.065 | 0.179 | 0.0993 | 0.0599 | 0.0692 |

- **Tab3;** Values of serum levels of testosterone in different groups;

| Testosterone | control | vehicle | diseased | metformin | bromelain | combined |
| --- | --- | --- | --- | --- | --- | --- |
| R1 | 122.5 | 102.3 | 801.4 | 555 | 525.3 | 332.6 |
| R2 | 190.4 | 125.9 | 928.9 | 701.8 | 683.4 | 439.5 |
| R3 | 105.2 | 240.7 | 547.3 | 342.7 | 308 | 107.9 |
| R4 | 215.7 | 191.3 | 744.1 | 546.7 | 500.8 | 300 |
| R5 | 265.9 | 370 | 859.5 | 661.2 | 619.4 | 405.4 |
| R6 | 98.9 | 278.3 | 1050 | 840.2 | 802.8 | 611.2 |
| R7 | 351.3 | 89.5 | 700.4 | 507.4 | 458.4 | 381.9 |
| R8 | 84.9 | 113.8 | 889.6 | 693.5 | 661.2 | 443.2 |

- **Tab4;** Values of serum levels of FSH in different groups;

| FSH | control | vehicle | diseased | metformin | bromelain | combined |
| --- | --- | --- | --- | --- | --- | --- |
| R1 | 0.507 | 0.149 | 0.919 | 0.121 | 0.299 | 0.037 |
| R2 | 0.813 | 0.375 | 2.001 | 0.921 | 1 | 0.94 |
| R3 | 0.407 | 0.484 | 1.524 | 0.51 | 0.71 | 0.595 |
| R4 | 0.301 | 0.577 | 2.911 | 1 | 1.21 | 1.478 |
| R5 | 0.432 | 1.001 | 3.431 | 1.53 | 1.67 | 1.599 |
| R6 | 0.232 | 0.811 | 2.412 | 1.71 | 1.711 | 1.167 |
| R7 | 0.872 | 0.677 | 3.021 | 2 | 2.1 | 1.811 |
| R8 | 1.544 | 0.73 | 1.121 | 0.4 | 0.81 | 0.498 |

- **Tab5;** Values of serum levels of LH in different groups;

| LH | control | vehicle | diseased | metformin | bromelain | combined |
| --- | --- | --- | --- | --- | --- | --- |
| R1 | 0.81488 | 0.31587 | 3.2595 | 2.458 | 1.075 | 0.0211 |
| R2 | 1.5795 | 0.64312 | 5.3907 | 4.561 | 2.983 | 1.721 |
| R3 | 1.1521 | 0.96714 | 4.369 | 3.541 | 2.031 | 1.123 |
| R4 | 0.70261 | 1.3541 | 6.2133 | 5.451 | 3.875 | 2.9635 |
| R5 | 0.92189 | 1.9021 | 9.4711 | 8.432 | 6.791 | 5.279 |
| R6 | 0.46148 | 2.0064 | 5.9872 | 4.991 | 3.534 | 2.179 |
| R7 | 1.8254 | 1.5609 | 7.332 | 6.475 | 5 | 3.221 |
| R8 | 2.5072 | 1.0067 | 4.5412 | 3.456 | 1.989 | 0.564 |

- **Tab6;** Values of FSH/LH ratio in different groups;

| FSH/LH | control | vehicle | diseased | metformin | bromelain | combined |
| --- | --- | --- | --- | --- | --- | --- |
| R1 | 0.622 | 0.474 | 0.0492 | 0.092 | 0.345 | 0.499 |
| R2 | 0.515 | 0.583 | 0.1218 | 0.21 | 0.299 | 0.322 |
| R3 | 0.354 | 0.5 | 0.144 | 0.199 | 0.234 | 0.377 |
| R4 | 0.429 | 0.426 | 0.1835 | 0.258 | 0.301 | 0.456 |
| R5 | 0.469 | 0.526 | 0.1815 | 0.099 | 0.367 | 0.344 |
| R6 | 0.503 | 0.404 | 0.343 | 0.389 | 0.399 | 0.555 |
| R7 | 0.478 | 0.434 | 0.3089 | 0.342 | 0.42 | 0.399 |
| R8 | 0.617 | 0.725 | 0.116 | 0.331 | 0.198 | 0.289 |

- **Tab7;** Values of serum levels of fasting glucose in different groups;

| glucose | control | vehicle | diseased | metformin | bromelain | combined |
| --- | --- | --- | --- | --- | --- | --- |
| R1 | 62.5 | 60.1 | 180.3 | 60 | 100 | 50.1 |
| R2 | 74.3 | 71.3 | 201 | 80 | 120 | 70 |
| R3 | 80.2 | 85.2 | 226.3 | 100 | 160 | 90 |
| R4 | 95.9 | 94.5 | 250.2 | 110 | 190 | 106 |
| R5 | 102.1 | 105.3 | 300.3 | 180 | 230 | 165 |
| R6 | 55.6 | 56.2 | 241.2 | 120 | 220 | 110 |
| R7 | 89.7 | 80.1 | 198.5 | 70 | 110 | 60 |
| R8 | 111 | 100 | 271.2 | 140 | 200 | 135.4 |

- **Tab8;** Values of serum levels of fasting insulin in different groups;

| insulin | control | vehicle | diseased | metformin | bromelain | combined |
| --- | --- | --- | --- | --- | --- | --- |
| R1 | 92.4 | 98.3 | 271.2 | 105 | 180 | 100 |
| R2 | 112.3 | 110.3 | 397.3 | 210 | 295 | 200 |
| R3 | 124.4 | 120.8 | 321.5 | 140 | 225 | 130 |
| R4 | 81.4 | 101.4 | 240.8 | 90 | 150 | 80 |
| R5 | 136.4 | 139.4 | 309.3 | 130 | 193 | 120 |
| R6 | 184.5 | 190.3 | 365.4 | 190 | 265 | 170 |
| R7 | 220 | 231.3 | 350.1 | 180 | 220 | 180 |
| R8 | 47.2 | 41.5 | 451.3 | 220 | 326 | 200 |

- **Tab9;** Values of HOMA-IR in different groups;

| HOMA-IR | control | vehicle | diseased | metformin | bromelain | combined |
| --- | --- | --- | --- | --- | --- | --- |
| R1 | 2.16 | 2.21 | 18.29 | 5.084 | 8.05 | 2.27 |
| R2 | 3.12 | 2.94 | 29.88 | 11.13 | 15.07 | 6.37 |
| R3 | 3.74 | 3.85 | 27.21 | 9.74 | 14.84 | 5.4 |
| R4 | 2.92 | 3.58 | 22.54 | 7.2 | 10.98 | 4.24 |
| R5 | 5.21 | 5.49 | 34.74 | 13.26 | 18.41 | 9.496 |
| R6 | 3.84 | 3.998 | 32.97 | 12.92 | 17.42 | 8.46 |
| R7 | 7.38 | 6.93 | 26 | 7.71 | 11.64 | 4.29 |
| R8 | 1.96 | 1.55 | 45.799 | 20.98 | 26.56 | 14.55 |

- **Tab10;** Values of tissue levels of TNFα in different groups;

| TNFα | control | vehicle | diseased | metformin | bromelain | combined |
| --- | --- | --- | --- | --- | --- | --- |
| R1 | 322.7 | 248.4 | 957.9 | 700 | 601 | 400 |
| R2 | 225.2 | 345.5 | 1070 | 802 | 705 | 510 |
| R3 | 270 | 410.2 | 895.7 | 622 | 532 | 311 |
| R4 | 380.1 | 109 | 1102 | 912 | 801 | 633 |
| R5 | 423.2 | 390.8 | 675.2 | 509.5 | 330 | 200 |
| R6 | 181.7 | 287.2 | 1020 | 807.9 | 645 | 501 |
| R7 | 503.5 | 195.8 | 1049 | 700 | 593 | 499 |
| R8 | 105.6 | 480.6 | 857.8 | 601 | 511 | 391 |

- **Tab11;** Values of tissue levels of SIRT1 in different groups;

| SIRT-1 | control | vehicle | diseased | metformin | bromelain | combined |
| --- | --- | --- | --- | --- | --- | --- |
| R1 | 45.22 | 46.32 | 22.11 | 23.54 | 20.32 | 25.12 |
| R2 | 34.22 | 35.21 | 16.69 | 26.21 | 24.12 | 28.88 |
| R3 | 30.21 | 31.28 | 17.24 | 29.51 | 28.54 | 30.21 |
| R4 | 35.32 | 36.15 | 12.56 | 32.15 | 16 | 35.32 |
| R5 | 38.54 | 39.94 | 19.22 | 20.1 | 18.3 | 38.54 |
| R6 | 20.41 | 21.43 | 10.87 | 35.14 | 30 | 34.66 |
| R7 | 32.21 | 33.09 | 15.43 | 23.14 | 26.54 | 20.41 |
| R8 | 36.33 | 37.29 | 13.45 | 27.3 | 21.42 | 32.21 |

**Vaginal smear stages;**

|  |  | |  |  |  |  |  |  |  |  |
| --- | --- | --- | --- | --- | --- | --- | --- | --- | --- | --- |
| days |  | 1 | | 2 | 3 | 4 | 5 | 6 | 7 |  |
|  | R1 | Estrus | | Metestrus | Diestrus | Proestrus | Estrus | Metestrus | Diestrus |  |
|  | R2 | Metestrus | | Diestrus | Proesrtus | Estrus | Metestrus | Diestrus | Proesrtus |  |
| control | R3 | Diestrus | | Proesrtus | Estrus | Metestrus | Diestrus | Diestrus | Proesrtus |  |
|  | R4 | Proesrtus | | Estrus | Metestrus | Diestrus | Diestrus | Proesrtus | Estrus |  |
|  | R5 | Estrus | | Metestrus | Diestrus | Proesrtus | Estrus | Metestrus | Diestrus |  |
|  | R6 | Diestrus | | Diestrus | Proesrtus | Estrus | Metestrus | Diestrus | Diestrus |  |
|  | R7 | Metestrus | | Diestrus | Proesrtus | Estrus | Metestrus | Diestrus | Proesrtus |  |
|  | R8 | Proesrtus | | Estrus | Metestrus | Diestrus | Diestrus | Proesrtus | Estrus |  |
|  |  |  | |  |  |  |  |  |  |  |
|  | R1 | Diestrus | | Proesrtus | Estrus | Metestrus | Diestrus | Diestrus | Proesrtus |  |
| vehicle | R2 | Metestrus | | Diestrus | Diestrus | Proesrtus | Estrus | Metestrus | Diestrus |  |
|  | R3 | Metestrus | | Diestrus | Diestrus | Proesrtus | Estrus | Metestrus | Diestrus |  |
|  | R4 | Diestrus | | Diestrus | Proesrtus | Estrus | Metestrus | Diestrus | Diestrus |  |
|  | R5 | Proesrtus | | Estrus | Metestrus | Diestrus | Proesrtus | Estrus | Metestrus | |
|  | R6 | Estrus | | Metestrus | Diestrus | Proestrus | Estrus | Metestrus | Diestrus |  |
|  | R7 | Proesrtus | | Estrus | Metestrus | Diestrus | Proesrtus | Estrus | Metestrus | |
|  | R8 | Diestrus | | Diestrus | Proesrtus | Estrus | Metestrus | Diestrus | Proesrtus |  |
|  |  |  | |  |  |  |  |  |  |  |
|  | R1 | Diestrus | | Diestrus | Diestrus | Diestrus | Diestrus | Diestrus | Diestrus |  |
|  | R2 | Diestrus | | Diestrus | Diestrus | Diestrus | Diestrus | Diestrus | Diestrus |  |
| diseased | R3 | Diestrus | | Diestrus | Diestrus | Diestrus | Diestrus | Diestrus | Diestrus |  |
|  | R4 | Diestrus | | Diestrus | Diestrus | Diestrus | Diestrus | Diestrus | Diestrus |  |
|  | R5 | Diestrus | | Estrus | Diestrus | Diestrus | Diestrus | Metestrus | Diestrus |  |
|  | R6 | Diestrus | | Diestrus | Diestrus | Diestrus | Diestrus | Diestrus | Proestrus |  |
|  | R7 | Diestrus | | Diestrus | Diestrus | Diestrus | Diestrus | Diestrus | Diestrus |  |
|  | R8 | Diestrus | | Diestrus | Diestrus | Diestrus | Metestrus | Diestrus | Diestrus |  |
